# Perceived impact of the menstrual cycle and hormonal contraceptives on physical exercise and performance in 1,086 athletes from 57 sports

**DOI:** 10.3389/fphys.2022.954760

**Published:** 2022-08-30

**Authors:** Linda Ekenros, Philip von Rosen, Guro Strøm Solli, Øyvind Sandbakk, Hans-Christer Holmberg, Angelica Lindén Hirschberg, Cecilia Fridén

**Affiliations:** ^1^ Department of Neurobiology, Care Sciences and Society, Division of Physiotherapy Karolinska Institutet, Huddinge, Sweden; ^2^ School of Sport Sciences, UiT, the Arctic University of Norway, Tromsø, Norway; ^3^ Department of Sports Science and Physical Education, Nord University, Bodø, Norway; ^4^ Department of Neuromedicine and Movement Science, Centre for Elite Sports Research, Norwegian University of Science and Technology, Trondheim, Norway; ^5^ Department of Health Sciences, Luleå University of Technology, Luleå, Sweden; ^6^ Department of Physiology and Pharmacology, Biomedicum C5, Karolinska Institutet, Stockholm, Sweden; ^7^ Department of Women’s and Children’s Health, Karolinska Institutet, Stockholm, Sweden; ^8^ Department of Gynecology and Reproductive Medicine, Karolinska University Hospital, Stockholm, Sweden

**Keywords:** amenorrhea, dysmenorrhea, hormones and athletes, premenstrual symptom, sex hormone

## Abstract

Female athletes train and compete under the potential influence of hormonal fluctuations during the menstrual cycle or during use of various hormonal contraceptives. Dysmenorrhea and premenstrual symptoms are common in the general population, as well as side effects of hormonal contraceptives. More detailed knowledge concerning prevalence and perceived impact of these symptoms on training and performance in different sports is required. The aim of the study was to 1) characterize perceptions of training and performance during the menstrual cycle phases and by hormonal contraceptive use in a large population of female athletes; 2) explore whether symptoms experienced are related to perceived performance; and 3) examine potential differences in these factors between athletes at different levels of performance. The study was based on self-reported data from 1,086 athletes from 57 sports at different performance levels *via* a web-based questionnaire. Thirty-seven percent (*n* = 407) of the athletes did not use hormonal contraceptives. In this group, menstrual cycle related symptoms were common across all athlete levels, particularly dysmenorrhea (74%, *n* = 300) and premenstrual symptoms (78%, *n* = 318), which also influenced perceived performance of aerobic fitness, muscle strength, mental sharpness, balance, and sleep quality. Sixty-three percent (*n* = 679) of the athletes used various hormonal contraceptives and 40% (*n* = 272) perceived a variety of side-effects. Physical performance was experienced equally independent of time-point of the pill-chart except for the period of inactive pills, which was associated with more negative impact. Nonetheless, only 18% (*n* = 191) of the athletes considered menstrual cycle or hormonal contraceptive issues when planning their training or competitions. These results indicate that greater focus is needed to identify and effectively treat different menstrual cycle and hormonal contraceptive related symptoms on an individual level.

## 1 Introduction

Female athletes train and compete under the potential influence of hormonal fluctuations during the menstrual cycle (MC) or during use of various hormonal contraceptives (HCs). Other female athletes may have menstrual disturbances and amenorrhea, which are more common among athletes compared to the general population (Redman and Loucks, 2005; Torstveit and Sundgot-Borgen, 2005; Nattiv et al., 2007). Although such hormonal impact may exert a significant influence on training and physical performance, recent studies request more knowledge in the field (Costello, Bieuzen and Bleakley, 2014; Bruinvels et al., 2017; Janse DE Jonge, Thompson and Han, 2019; Findlay et al., 2020; Cowley et al., 2021). Specifically, there is a lack of sufficiently large studies reporting results on sub-groups at different levels of performance both without and with different types of HCs.

The MC can be divided into three specific phases: the follicular phase (menses to ovulation), the ovulatory phase (at mid-cycle and lasts 24–48 h), and the luteal phase (from ovulation to next menses) (Hirschberg, 2022). Although the levels of estradiol and progesterone vary significantly during these phases, previous research investigating the effect of the MC on physical performance has shown varying results (Giacomoni et al., 2000; Oosthuyse, Bosch and Jackson, 2005; Bushman, Masterson and Nelsen, 2006; Shaharudin, Ghosh and Ismail, 2011; Vaiksaar et al., 2011; Julian et al., 2017; Barba-Moreno et al., 2019; McNulty et al., 2020; Taipale-Mikkonen et al., 2021). A recent meta-analysis concluded that muscle strength, as well as maximal and submaximal aerobic performance, are trivially reduced during the early follicular phase (during menses, low levels of estradiol, and progesterone), compared with all other MC phases (McNulty et al., 2020). However, the meta-analysis emphasized that general guidelines on the effects of the MC on exercise performance can yet not be formulated due to methodological limitations of the included studies (McNulty et al., 2020). Most studies were underpowered, lacking clear definition of cycle phase and have not considered dysmenorrhea, variation in general well-being or premenstrual symptoms (PMS) during the cycle. There is a lack of studies investigating menstrual related symptoms on self-reported effects of muscle strength, aerobic fitness, mental sharpness, balance, and sleep quality in a large group of athletes in different sports and performance levels.

The use of HCs in the general population of Scandinavian women of reproductive age is approximately 40% (Lindh et al., 2017). A higher proportion, 50%–57%, of HC use has been reported for different athlete populations in various European countries (Martin et al., 2018; Oxfeldt et al., 2020; Solli et al., 2020; Nolan, Elliott-Sale and Egan, 2022), and Engseth et al. (Engseth et al., 2022) showed a proportion of 68% in Norwegian cross-country skiers and biathletes. In general, combined oral contraceptives (OCs) containing estrogen and progestin are reported as more commonly used (60%–74% of athletes) in athletes compared to progestin-only HCs (Martin et al., 2018; Oxfeldt et al., 2020; Solli et al., 2020). However, the Norwegian study showed inverse proportions with 36% using combined OCs and 64% progestin-only (Engseth et al., 2022). A recent meta-analysis by Elliott-Sale and colleagues (2020) concluded that combined OCs may slightly reduce exercise performance compared to non-HC use (Elliott-Sale et al., 2020). Hypothetically, this might at least partly be related to side effects of HCs including mood changes (Zethraeus et al., 2016; Lundin et al., 2017; Ekenros et al., 2019). The influence of various HCs on physical performance in athletes at different competition levels has earlier been studied in cross-country skiers and biathletes (Engseth et al., 2022), but data from other sports are currently lacking.

Accordingly, more detailed knowledge concerning the perceived impact of the MC and different administration of HCs on the training and performance of female participants in different sports and performance levels is required. Therefore, the current investigation was designed to 1) characterize perceptions of training and performance during the MC phases and by HC use in a large population of female athletes; 2) explore whether symptoms experienced during different phases of the MC and HC use are related to perceived performance; and 3) examine potential differences in these respects between athletes within different levels of performance.

## 2 Materials and methods

### 2.1 Study population and distribution of the survey

Female athletes at least 18 years of age and active members of the Swedish or Norwegian Sports Federation were recruited *via* social media and emails to professional sports clubs. All participants received information about the study and provided their written consent prior to completing the questionnaire. This study was approved by the Swedish Ethical Review Board (D.nr 2020-00418).

### 2.2 Design of the survey

A questionnaire was designed to answer the aim of the study. Based on a pilot study of the questionnaire, involving 124 female athletes, median age 20 [Interquartile range (IQR) 19–24 years], median body mass index 22.2 (IQR, 21.2–23.5), minor modifications regarding the description of cycle phases were made. To ensure uniform interpretation of the different phases of the MC, a picture was placed together with the questions related to these phases.

The questionnaire was open between June and December of 2021. The questionnaire consisted of 46 questions and required approximately 20 min to complete and was filled in once online by the participants. The questionnaire was distributed online to female athletes together with an invitation to participate and with a link to the questionnaire attached. Six of the questions were close-ended and 40 were multiple choice. Written responses were only requested when the answer chosen was “Other.” The participating athletes reported demographic information, training and competition level, menstrual history, and use of HCs as well as MC or HC-related symptoms such as dysmenorrhea, PMS and side-effects of HCs. This questionnaire was originally written in Swedish and later translated into Norwegian by a native speaker.

### 2.3 Definitions

#### 2.3.1 Athlete level

The athletes were categorized by competitive levels as top-elite athletes (i.e., athletes participating in the Olympic games, World or European championships; 15%, *n* = 158), elite athletes (i.e., athletes participating in other international competitions, national championship, or the highest national league; 36%, *n* = 393), and sub-elite athletes (i.e., athletes participating in the second highest national league or district competitions; 49%, *n* = 535).

#### 2.3.2 Normal menstrual cycle length

An average MC last for 28 days, with a between-individual variation of 23–35 days (Hirschberg, 2022).

#### 2.3.3 Early follicular phase

From the first day of menstrual bleeding, i.e., cycle day 1 to cycle day 7.

#### 2.3.4 Ovulation phase

Two days around ovulation.

#### 2.3.5 Late luteal phase

The premenstrual phase, from 1 week after ovulation until next menses.

#### 2.3.6 Oligomenorrhea

Menstrual cycle length of more than 36 days but less than 3 months (Nattiv et al., 2007).

#### 2.3.7 Primary amenorrhea

Absence of menarche in women 15 years or older (Nattiv et al., 2007).

#### 2.3.8 Secondary amenorrhea

Absence of menstrual bleeding for three or more consecutive months (Nattiv et al., 2007).

#### 2.3.9 Premenstrual symptoms

Cyclical symptoms of physical (bloating, breast tenderness, headache) mental and/or social character (absent minded, depression, fatigue, irritability, loss of concentration, loss of energy, tearfulness) that appears in the late luteal phase and disappear a few days after onset of menses (Angst et al., 2001).

### 2.4 Statistical analysis

Written answers were categorized. Categorical variables were expressed as percentages (%) and total numbers (*n*) and continuous variables as means and standard deviations (SD). The different groups (non-users and users of HCs; users of different types of HCs; athletes’ competitive levels) were compared using the independent samples *t*-test and one-way ANOVA, respectively, and the level of statistical significance set at <0.05. When significant difference was indicated, the Tukey post hoc test was applied for pairwise comparisons and standardized residuals was calculated for categorical data. The Bonferroni correction was applied to *p*-values. Effects sizes were determined as Cohen’s D values for differences in means and *w* indices for Chi-square tests or by applying the Eta-squared test to ANOVA models. Bootstrap percentile 95% confidence intervals (95% CI) were constructed based on drawing and replacing 1,000 bootstrap replicates, to compare differences on physical and mental performance and sleep quality for the different phases of the MC and at different time points in the OC-pill chart. Subgroup analyses, using the Chi-square test, were performed to compare differences in menstrual related pain across athletes with different perceptions of physical performance during the early follicular phase and to compare differences in PMS across athletes with different perceptions of physical performance during the late luteal phase. All analyses were performed utilizing Microsoft Excel (Microsoft Corporation, Redmond; WA, United States) and the IBM Statistical Package for the Social Sciences (version 27.0, SPSS Inc., Chicago, IL, United States).

## 3 Results

**FIGURE 1:**
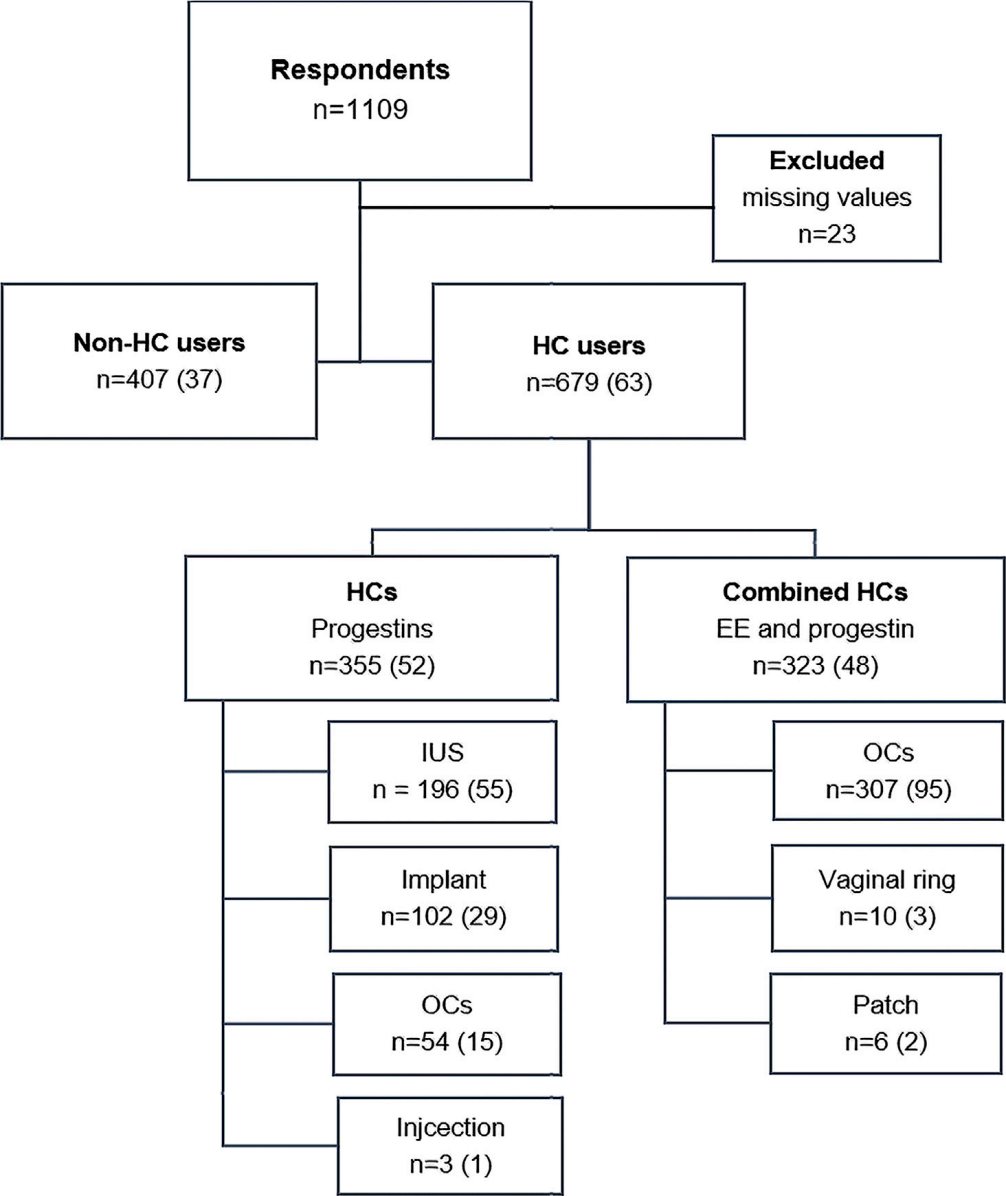
Division of the respondent athletes into users and non-users of hormonal contraceptives (HC users). Each box indicates the number (*n*) of respondents as well as the percentage (%) of the total number. EE = ethinyl estradiol; IUS = intrauterine system; OCs = oral contraceptives.

Of 1,109 respondents, 1,086 athletes from 57 different sports (Table 1) consented to participate in the study. Those 1,086 athletes were divided into non-users (37%, *n* = 407) and HC users (63%, *n* = 679) (Figure 1) and their demographic characteristics are documented in Table 2.

**TABLE 1:** The number (percentage) of respondents participating in different sports.

Soccer	312 (29)	Skateboarding	4 (<1)
Handball	243 (22)	Weightlifting	4 (<1)
Orienteering	94 (9)	Curling	3 (<1)
Cross-country skiing	58 (5)	Gym training	3 (<1)
Floorball	42 (4)	Judo	3 (<1)
Swimming	35 (3)	Badminton	2 (<1)
Gymnastics	31 (3)	Beach volleyball	2 (<1)
Triathlon	24 (2)	Canoeing (sprint)	2 (<1)
Powerlifting	19 (2)	CrossFit	2 (<1)
Cycling	17 (2)	Snowboarding	2 (<1)
Budo	16 (1)	Ski orienteering	2 (<1)
Alpine skiing	15 (1)	Tennis	2 (<1)
Biathlon	15 (1)	Equestrian vaulting	2 (<1)
Figure skating	14 (1)	Wrestling	2 (<1)
Athletics (sprint/jump)	12 (1)	American football	1 (<1)
Athletics (distance running^a^)	13 (1)	Aerobics	1 (<1)
Canoeing (distance)	9 (1)	Bandy	1 (<1)
Basketball	8 (1)	Enduro	1 (<1)
Equestrian	8 (1)	Free skiing	1 (<1)
Ice hockey	8 (1)	Drill	1 (<1)
Archery	7 (1)	Golf	1 (<1)
Roller derby	7 (1)	Kick boxing	1 (<1)
Athletics (discus, javelin)	6 (1)	Rhythmic gymnastics	1 (<1)
Ski jumping	6 (1)	Shooting	1 (<1)
Volleyball	6 (1)	Skicross	1 (<1)
Climbing	4 (<1)	Taekwondo	1 (<1)
Rowing	4 (<1)	Windsurfing	1 (<1)
Rugby	4 (<1)	Not indicated	3 (<1)

a middle-/long-distance running.

**TABLE 2:** Demographic characteristics of all respondents and of the sub-groups of non-users and users of hormonal contraceptives (HC).

Characteristic	All (*n* = 1,086)	Non-users (*n* = 407)	Users (*n* = 679)	*p*-value	Effect size
Age, years, mean (SD)	24.1 (6.8)	24.6 (7.8)	23.8 (64)	0.053^a^	*d* = 0.11
Training hours per week, mean (SD)	10.4 (5.6)	10.5 (5.2)	10.4 (5.7)	0.830	*d* = 0.02
Height, cm, mean (SD)	169 (6.9)	170 (7.0)	169 (6.8)	0.384	*d* = 0.02
Weight, kg, mean (SD)	66.7 (9.5)	66.0 (10)	67.1 (9.2)	0.040^a^	*d* = 0.01
Body mass index, kg/m^2^, mean (SD)	23.2 (2.9)	22.9 (3.0)	23.4 (2.8)	0.006^a^	*d* = 0.17
Menarche <11 years of age, *n* (%)	35 (3)	15 (4)	20 (3)	0.504	*w* = 0.02
Menarche at 11–16 years of age, *n* (%)	1,007 (93)	372 (91)	635 (94)	0.193	*w* = 0.04
Menarche >16 years of age, *n* (%)	26 (2)	12 (3)	14 (2)	0.355	*w* = 0.03
Dysmenorrhea, *n* (%)	742 (68)	300 (74)	441 (65)	0.003^a^	*w* = 0.09
Regular menstrual bleeding	n.a	305 (74)	n.a	n.a	n.a
History of secondary amenorrhea, *n* (%)	432 (40)	141 (35)	291 (43)	0.007^a^	*w* = 0.08
Current amenorrhea, *n* (%)	n.a	40 (10)	n.a	n.a	n.a

Means (SD) and numbers (%). The *p*-values for comparisons of non-users and users of HCs, were calculated using an independent sample *t*-test and one-way ANOVA., Effect sizes are calculated as Cohen´s d-values (*d*) or the *w* index. n.a = not applicable.

a Indicated a significant difference.

Most of the athletes experienced menarche within normal age range (93%), Table 2. Dysmenorrhea was reported by 68% (n = 742) of all the athletes. Of those, 72% (*n* = 534) reported to use medication for pain relief. The use of pain relief without prescription was most frequently reported, by 65% (*n* = 485) of the athletes, pain relief with prescription was used by 7% (*n* = 50), and 13% (*n* = 99) of the athletes reported use of OCs as treatment for dysmenorrhea. The non-HC users experienced dysmenorrhea significantly more frequent than the HC users (*p* = 0.003, *w* = 0.09). On the other hand, a history of secondary amenorrhea was reported more frequently by HC users than non-users (43% vs. 35%). Of those with previous amenorrhea, 32% (*n* = 139) and 29% (*n* = 128) related their amenorrhea to increased amount of training or weight loss, respectively.

Out of all the athletes, 76% (*n* = 824) believed that their MC or use of HCs could have some or a high impact on their performance at training, and 73% (*n* = 791) on their competitive performance (partial or high impact). Despite this, only 18% (*n* = 191) of the athletes reported to partially or to a high extent scheduling their training based on their MC or use of HCs. Altogether, 45% (*n* = 494) of the athletes had refrained from planned training, and 14% (*n* = 151) from competition for reasons related to their MC or use of HCs (Table 3). The most common reason to refrain from training or competition was menstrual related pain (33%, *n* = 353 and 11%, *n* = 118, respectively). Generally, there was no significant difference between the non-HC users and HC users in refraining from training (48%, *n* = 196 and 44%, *n* = 298) *p* = 0.171, *w* = 0.04 or from competition (15%, *n* = 60 and 13%, *n* = 91), *p* = 0.532, *w* = 0.02 (Table 3). However, the non-HC users refrained significantly more frequent from training due to mental symptoms of PMS (e.g., depression, irritability, loss of concentration, tearfulness), compared to the HC users (7%, *n* = 27 and 4%, *n* = 26), *p* = 0.003, *w* = 0.06).

**TABLE 3:** The numbers (percentage) of respondents who reported refraining from training (*n* = 494) and/or competition (*n* = 151) for different reasons related to their menstrual cycle or usage of hormonal contraceptives. Premenstrual symptoms of mental kind were significantly more frequent reported in the non-users (*p* = 0.036) of HCs. Every athlete report one or two reasons to refrain from training or competition.

Reason	All		Non-users		Users		Training		Competition	
	Traning	Competition	Training	Competition	Traning	Competition	*p*-value	Effect size	*p*-value	Effect size
	*n* = 494, 45%	*n* = 151, 14%	*n* = 196, 48%	*n* = 60, 15%	*n* = 298, 44%	*n* = 91, 22%	0.171	*w =* 0.04	0.532	*w* = 0.02
Menstrual related pain, *n* (%)	353 (33)	118 (11)	141 (35)	43 (11)	212 (31)	75 (11)	0.244	*w* = 0.02	0.118	w = 0.13
Fatigue, *n* (%)	60 (17)	13 (2)	33 (8)	7	27 (7)	6	0.805	w = 0.01	0.286	w = 0.01
PMS of mental kind ¤, *n* (%)	53 (5)	19 (2)	27 (7)	8	26 (4)	11 (2)	0.036*	w = 0.06	0.821	w < 0.01
Menorrhagia, *n* (%)	40 (4)	10 (1)	12 (3)	3	28 (4)	7	0.320	w = 0.02	0.515	w = 0.05
Nausea, *n* (%)	29 (3)	16 (1)	12 (3)	8	17 (3)	8	n.a	n.a	n.a	n.a
Sickness, *n* (%)	16 (1)	6	4	2	12 (2)	4	n.a	n.a	n.a	n.a
Migraine, *n* (%)	14 (1)	6	6	3	8	3	n.a	n.a	n.a	n.a
Discomfort, *n* (%)	15 (1)	2	8	1	7	1	n.a	n.a	n.a	n.a
Dizziness, *n* (%)	9	4	5	0	4	4	n.a	n.a	n.a	n.a
Poor sleep quality, *n* (%)	7	1	3	0	4	1	n.a	n.a	n.a	n.a
Diarrhea, *n* (%)	4	2	2	0	2	1	n.a	n.a	n.a	n.a
Inability to use a tampon, *n* (%)	3	1	2	1	4	0	n.a	n.a	n.a	n.a
Urinary incontinence, *n* (%)	3	1	0	0	2	1	n.a	n.a	n.a	n.a

¤ PMS, of mental kind = depression, irritability, loss of control, tearfulness. * Indicated a significant difference. n.a = not applicable, due to low values.

### 3.1 Impact of the menstrual cycle

**FIGURE 2:**
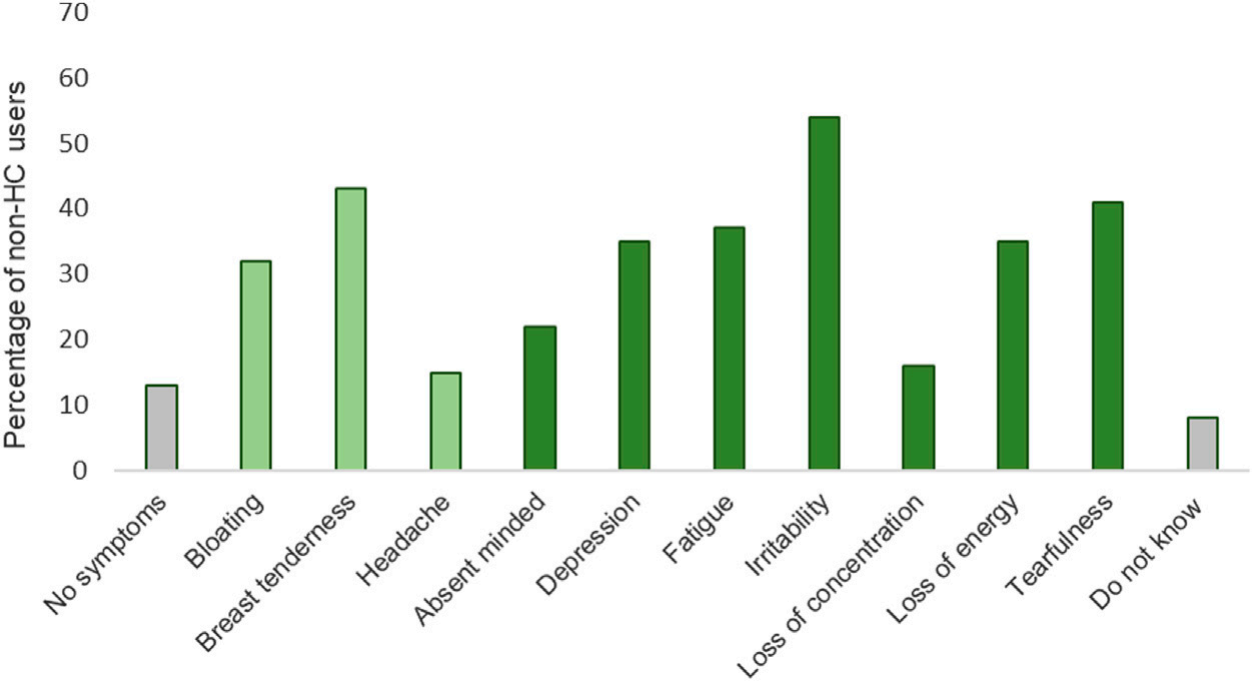
Premenstrual symptoms reported by the non-users (*n* = 407) of hormonal contraceptives. Physical symptoms in light green and mental symptoms in green. Breast tenderness was the most frequent reported physical symptom (43%, *n* = 172) and irritability the most frequent reported mental symptom (54%, *n* = 214).

Most of the non-HC users, 52% (*n* = 210), reported the length of their MC to be 26–30 days, whereas 6% (*n* = 24) of the athletes reported a length of more than 36 days (oligomenorrhea) and 2% (*n* = 8) less than 23 days. Ten percent (*n* = 40) of the non-HC users reported an absence of menstrual bleeding > 3 months (secondary amenorrhea). In total, 78% (*n* = 318) of the non-users of HCs experienced PMS. The most common physical symptom was breast tenderness (43%, *n* = 172), and the most common mental symptom was irritability reported by 54% (*n* = 214) of the athletes (Figure 2). A significant proportion of the athletes reported 3-6 PMS (40%, *n* = 161), and 14% (*n* = 56) reported 7–10 symptoms.

**FIGURE 3:**
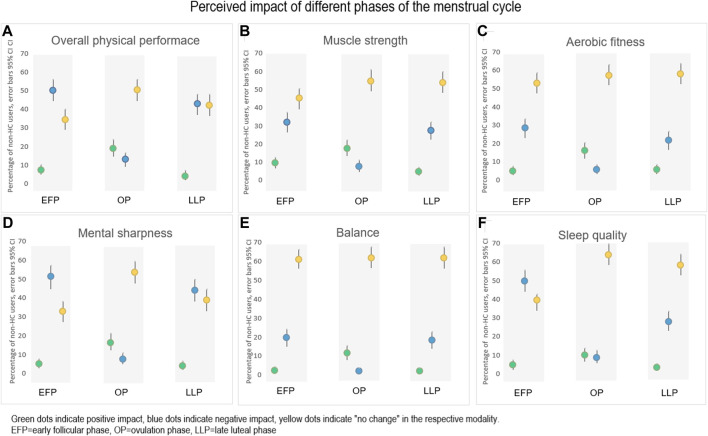
The perceived impact (positive, negative or no change) of **(A)** Overall physical performance, **(B)** Muscle strength, **(C)** Aerobic fitness, **(D)** Mental sharpness, **(E)** Balance and **(F)** Sleep quality, during different phases of the menstrual cycle (*n* = 299 in each phase). A proportion of 6%–24% of the athletes answered “I do not know” on perceived impact for the different modalities, respectively (data not shown).

The non-HC users reported a significant positive impact (*p* < 0.050) on their overall physical performance during the ovulation phase compared to the early follicular phase and late luteal phase respectively (Figure 3A). On the other hand, 50% (*n* = 149) and 43% (*n* = 129) of the athletes reported a significant negative impact (*p* < 0.050) on their physical performance during the early follicular phase and late luteal phase, respectively, compared to the ovulation phase (Figure 3A). Sub-analysis of overall physical performance showed that menstrual related pain was reported significantly (*p* < 0.001; *w* = 0.29) more frequent by the athletes who perceived negative impact during the follicular phase (92%, *n* = 135), compared to those who reported no impact or positive impact (70%, *n* = 87), respectively. During the late luteal phase, 100% (*n* = 134) of the athletes that perceived negative impact of overall physical performance experienced PMS, compared to the 76% (*n* = 105) of the athletes that reported positive or no impact (*p* < 0.001, *w* = 0.35) (Figure 3A).

A similar pattern of a positive impact during the ovulation phase and a negative impact during the early follicular phase and the late luteal phase was also shown in muscle strength, aerobic fitness, balance, and sleep quality (Figures 3B,C,E,F). Menstrual related pain was significantly (*p* < 0.001, *w* = 0.32) more frequently reported by the athletes that perceived negative impact on mental sharpness during the early follicular phase (93%, *n* = 140), compared to 69% (*n* = 78) of those who reported positive or no impact on mental sharpness. For the perceived negative impact on mental sharpness during the late luteal phase, 98% (*n* = 126) of the athletes reported PMS compared to the 72% (*n* = 92) of those who perceived a positive or no impact (*p* < 0.001, *w* = 0.37) (Figure 3D).

### 3.2 Impact of hormonal contraceptive use

As shown in Figure 1, HCs with progestins only were the most common type of HCs. Of combined methods, oral contraceptives of combined type (OCs) were the most common method used among the athletes. Different brands of combined OCs and doses of synthetic ethinyl estradiol and progestins are presented in Supplementary Table 1. Of those using combined OCs, 64% (*n* = 196) were using progestin dominant (second generation) and 18% (*n* = 56) estrogen dominant (third generation) (Shahnazi et al., 2014), and the rest combined OCs that were not classified. In the sub-group of combined OC-users, 24% (*n* = 75) reported intake of both active and inactive pills. Forty-seven percent (*n* = 144) reported to regularly, and 28% (*n* = 87) to occasionally skip the inactive pills to avoid bleeding. Birth control was the primary reason for HC use and were reported by 82% (*n* = 557) of the athletes. Other reasons for HC use were treatment of dysmenorrhea (41%, *n* = 278), to postpone menstrual bleeding (31%, *n* = 210), and as treatment for irregular MCs (16%, *n* = 109).

**FIGURE 4:**
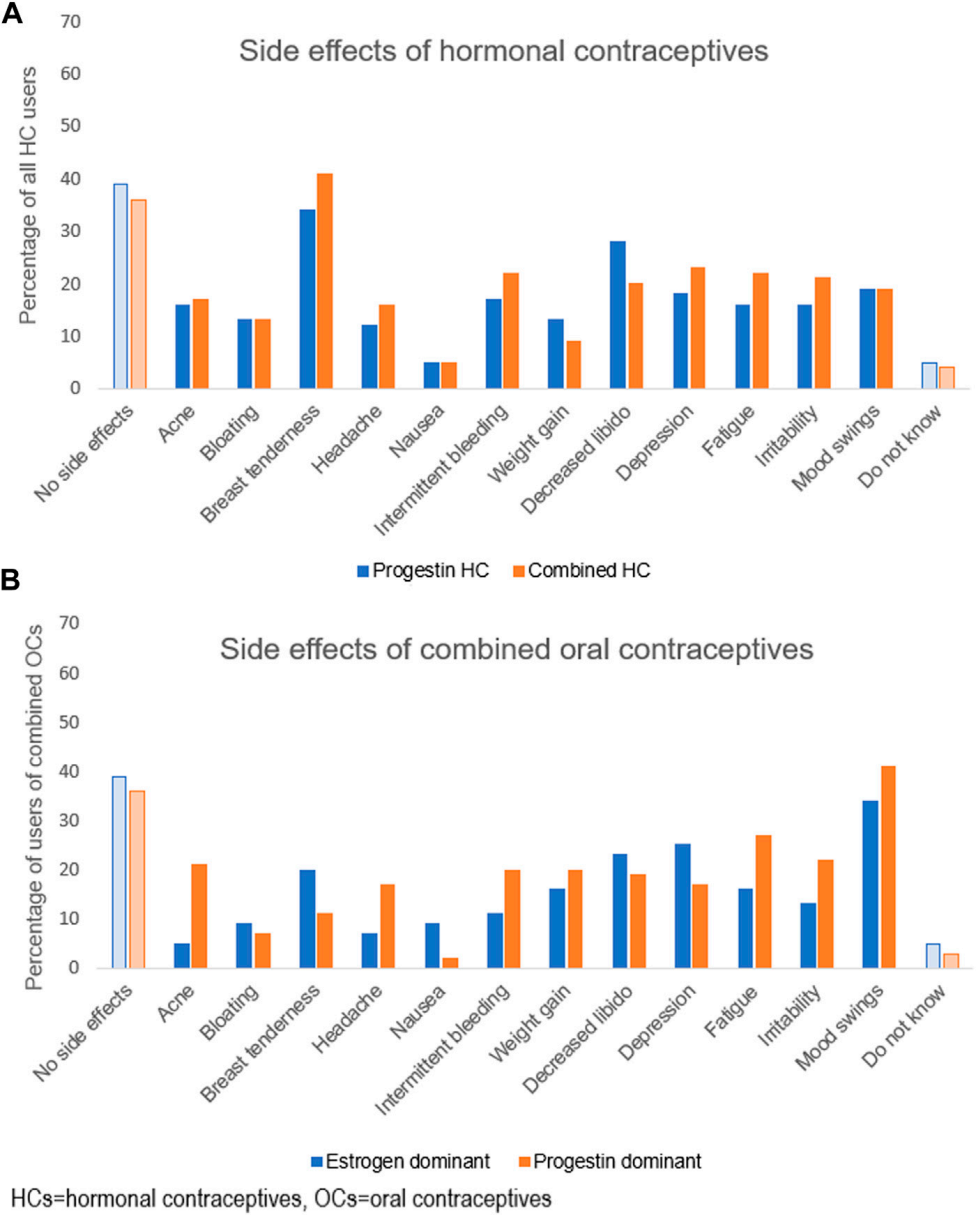
Side effects experienced by the athletes using hormonal contraceptives, in percentage. In **(A)** side effect of hormonal contraceptives containing progestins alone (49%, *n* = 299) and hormonal contraceptives containing a combination of progestins and ethinyl estradiol (51%; *n* = 316). A total of 615 (91%) users of hormonal contraceptives answered this question. In **(B)** side effects of combined oral contraceptives, divided into estrogen dominant oral contraceptives (18%; *n* = 56) and progestin dominant oral contraceptives (51%; *n* = 156). The oral contraceptives of non-specific dominance (31%; *n* = 95) (data not shown).

Different side effects of HCs were reported by 40% (*n* = 269) of the athletes and there was no significant difference between progestins only and combined HCs. Breast tenderness followed by decreased libido were the most frequent side effects reported (Figure 4A). The most frequent side effect of combined OCs was mood swings, reported by 41% (*n* = 128) and 34% (*n* = 103) of the users of progestin and estrogen dominant OCs, respectively (Figure 4B). The perceived side effects among the users of combined OCs were in general similar between progestin dominant and estrogen dominant OCs.

**FIGURE 5:**
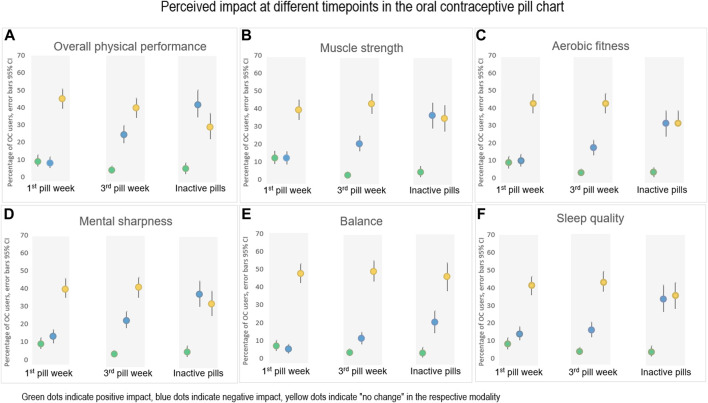
The perceived impact (positive, negative or no change) of **(A)** Overall physical performance, **(B)** Muscle strength, **(C)** Aerobic fitness, **(D)** Mental sharpness, **(E)** Balance, and **(F)** Sleep quality, during different time points in the pill chart of combined hormonal contraceptives. First and third pill week (*n* = 307) and at the time point for inactive pills (*n* = 163). A proportion of 18%–46% of the athletes answered “I do not know” on perceived impact for the different modalities, respectively (data not shown).

Overall, 47% (*n* = 144) of the athletes using combined OCs reported a perception of variation in their physical performance at different time points of the OC pill chart. In general, the athletes perceived the most negative impact during the inactive phase of the pill chart. Significantly negative impact was reported in all aspects of physical and mental performances, as well as in sleep quality during the inactive pills compared to during the first and the third pill week (*p* > 0.05) Figures 5A–F. Dysmenorrhea was reported by 77% (n = 52) of the athletes who perceived a negative impact on their overall physical performance during the time point of inactive pills (Figure 5A).

### 3.3 Comparisons based on athlete levels

The demographic characteristics of the athletes competing at different levels are presented in Table 4. There was an overall significant difference in average age (*p* < 0.001, *d* = 0.03) and weekly hours of training (*p* < 0.001, *d* = 0.27) between the competition levels. The sub-elite athletes had a significantly (*p* < 0.001, *d* = 0.02) higher body mass index compared to the top-elite and elite athletes. Regular menstrual bleeding was reported to a significantly less extent (*p* = 0.036, *w* = 0.08) in the elite athletes compared to those at the other two levels (top-elite and sub-elite). The frequency of dysmenorrhea in non-HC users was comparable for the different athlete levels (*p* = 0.174, *w* = 0.06).

**TABLE 4:** Demographic characteristics of the athletes’ competitive levels. Top-elite: Olympic games, World championships, and/or European championships. Elite: other international competitions, Swedish or Norwegian championships, the highest national league. Sub-elite: the next highest national league, and district competitions.

Characteristic	Top-elite (*n* = 158)	Elite (*n* = 393)	Sub-elite (*n* = 535)	*p*-value	Effect size
Age in years, mean (SD)	26.7 (6.2)^a^	22.8 (5.8)^a^	24.4 (7.5)^a^	<0.001	*d* = 0.03
Body mass index, kg/m2, mean (SD)	22.6 (2.6)	22.9 (2.7)	23.6 (3.0)^a^	<0.001	*d* = 0.02
Average training, hours/week, mean (SD)	16.6 (7.8)^a^	11.1 (4.5)^a^	8.1 (3.6)^a^	<0.001	*d* = 0.27
History of secondary amenorrhea, *n* (%)	70 (44)	165 (42)	197 (37)	0.129	*w* = 0.06

Non-users of HCs, *n* (%)	71 (45)	160 (41)	178 (33)^a^	0.009	*w* = 0.09
Regular menstrual bleeding	57 (80)	111 (69)^a^	137 (77)	0.036	*w* = 0.08
Current secondary amenorrhea	7 (10)	20 (13)	13 (7)	0.090	*w* = 0.07
Dysmenorrhea	50 (71)	116 (73)	135 (76)	0.174	*w* = 0.06

Users of HCs, *n* (%)	87 (55)	233 (59)	357 (67)^a^	0.009	*w* = 0.09
Progestins only	46 (53)	117 (50)	191 (54)	0.098	*w* = 0.07
Combined HCs	41 (47)	116 (50)	166 (46)	0.468	*w* = 0.04
Dysmenorrhea	49 (56)	148 (64)	243 (68)^a^	0.002	*w* = 0.11

a Significant difference as indicated by the Tukey post-hoc test for ANOVA, and by residuals and Bonferroni corrections for the Chi-squared test. The effect sizes were calculated utilizing the Eta-squared and Cohen´s D tests.

HCs were used to a significantly (*p* < 0.001, *d* = 0.10) higher proportion in the sub-elite athletes compared to the other two levels (Table 4). Dysmenorrhea was significantly (*p* = 0.005, *w* = 0.15) more frequently reported among HC-users in the sub-elite athletes than those of the other two athlete levels. HCs as treatment of dysmenorrhea was significantly (*p* < 0.001, *w* = 0.20) more common in the sub-elite athletes compared to the other two levels.

Forty-three percent (*n* = 68), 46% (*n* = 179), and 44% (*n* = 237) of the athletes at the top elite level, elite and sub-elite, respectively, had refrained from training due to symptoms related to menstruation or use of HCs (*p* = 0.910, *w* = 0.01). Moreover, 11% (*n* = 17), 16% (*n* = 61), and 14% (*n* = 73), respectively, reported refraining from competition because of such symptoms, with no significant difference between the levels (*p* = 0.585, *w* = 0.03).

## 4 Discussion

This is by far the largest study reporting symptoms related to the MC and HC use in athletes and their perceived impact on physical performance at different levels of performance. We found that symptoms related to the MC are common across all athlete levels, particularly dysmenorrhea and PMS which also seem to influence perceived performance of aerobic fitness, muscle strength, mental sharpness, balance, and sleep quality. A large proportion of the athletes (63%) used HC and perceived a variety of side-effects. However, performance was experienced equally independent of time-point of the pill-chart except for the period of inactive pills, which was associated with the most negative impact. Nonetheless, few of the athletes consider MC or HC issues when planning their training or competitions.

A MC length within normal range was reported by most of the non-HC users. However, ten percent of the athletes reported a current secondary amenorrhea and six percent reported their MC to be > 35 days, indicating oligomenorrhea. This agrees with previous research reporting that menstrual irregularities, such as amenorrhea and oligomenorrhea, is common among elite athletes, potentially due to relative energy deficiency (RED-S) and extensive training (Torstveit and Sundgot-Borgen, 2005; Nattiv et al., 2007; Joy et al., 2014).

The non-HC users in our study perceived a negative impact on several aspects of performance during the early follicular phase (menstrual bleeding) and during the late luteal phase, compared to the ovulation phase of the MC. The negative impact on performance was in turn showed to be related to menstrual pain during the early follicular phase and PMS during late luteal phase. This agrees with Martin et al. (2018) and Solli et al. (2020) who discussed a likely relationship between symptoms such as dysmenorrhea and/or PMS and an impact of performance during these phases.

Even though most of our athletes (74%) were affected by menstrual related pain, only one third reported that this pain led them to refrain from training. In comparison, Solli et al. (2020) found that 22% of their female athletes altered their training repeatedly due to negative effects of the MC, while Martin et al. (2018) reported a corresponding frequency of only 4%. A related qualitative study concluded that although female athletes’ express acceptance of menstrual related symptoms, they do not consider such symptoms to be sufficient reason to refrain from training and are reluctant to discuss their symptoms with their coaches (Findlay et al., 2020).

There are effective medical treatments for pain relief of dysmenorrhea. The first choice of treatment regimen is non-steroid anti-inflammatory drugs (NSAID) and/or hormonal suppression by combined OC treatment (Wong et al., 2009). To reduce the severity of symptoms, 52% of the athletes in the study by Solli et al. (2020) reported to have used painkillers. Data from our study show that 72% of the athletes with dysmenorrhea used medical treatment for pain relief and most common were different painkillers without and with prescription, 65% and 7% respectively. OCs as treatment for dysmenorrhea was used by 13% of the athletes. These results suggest that many athletes in our study did not use adequate treatment. However, there is a need for more knowledge of how different types of treatment regimens for dysmenorrhea affects the results of training and performance.

PMS is characterized by several MC-related symptoms, both somatic (breast tenderness, bloating, headache) and mood/mental symptoms (irritability, depression, tearfulness, fatigue) during the luteal phase of the MC (Angst et al., 2001). Among the 78% of our respondents who experienced PMS, irritability was the most frequent reported symptom. Overall, these female athletes perceived a negative impact on overall physical performance, aerobic fitness, muscle strength, mental sharpness, postural control, and quality of sleep in the late luteal phase of the MC.

A recent meta-analysis of studies involving objective determination of muscular and aerobic performance during different phases of MC, concluded that performance was only trivially decreased during the early follicular phase compared to the ovulatory and luteal phase (McNulty et al., 2020). Additionally, an altered postural control in the luteal phase has been observed (Darlington et al., 2001), especially in women experiencing PMS (Fridén et al., 2003; Fridén et al., 2005). It might be that the influence of the MC on physical performance is not primarily mediated *via* the hormonal changes during the different phases, but indirectly through MC-related symptoms like dysmenorrhea, PMS and reduced quality of sleep since these symptoms was related to performance among the athletes in our study.

The majority of the athletes (63%) in this study were using HCs, which is a higher proportion than among the general population (Lindh et al., 2017), as well as among other groups of elite athletes (Martin et al., 2018; Oxfeldt et al., 2020; Solli et al., 2020; Nolan, Elliott-Sale and Egan, 2022). However, Engseth et al. (2022) showed a similar proportion, 68%, in cross-country skiers and biathletes. Progestin-only HCs and combined HCs were equally common, with a slight predominance for progestins (52% vs. 48%, respectively). This contrasts with Martin et al. (2018) and Solli et al. (2020) reporting a higher proportion of combined HCs. The relatively high use of progestins in our study could be explained by the promotion of long-acting reversible contraception (LARC), including hormonal IUD and implants, in recent years in the Nordic countries to provide effective contraception for an extended period of time (Lindh et al., 2017) which also was reported by Furu et al. (2021) and Engseth et al. (2022). In agreement with earlier reports (Martin et al., 2018; Solli et al., 2020), oral administration of combined OCs was the most common combined method in our athletes compared to vaginal ring or patch.

In addition to birth control, OCs are utilized to treat dysmenorrhea, menorrhagia, amenorrhea, as well as to postpone or omit menstrual bleeding (Elliott-Sale et al., 2020), which was also the case among the athletes in our study. As expected, dysmenorrhea was significantly less frequently reported by HC users than non-HC users in our study. Back-to-back OC cycles (i.e., skipping the days of inactive pills) designed to avoid bleeding, were reported by 47% of our athletes. This is not surprising considering earlier observations on the application of this strategy to manipulate and alleviate symptoms related to menstrual bleeding (Martin et al., 2018; Schaumberg et al., 2018). Perceived side-effects of HC use were common and reported by 40% of the users. However, we found no significant difference in the prevalence or nature of side effects between those taking progestins only or combined HCs.

Almost half of the athletes using combined OCs reported perceived influence of OCs on physical performance. However, the general picture was a negative impact during the inactive phase of the pill chart when usually a withdrawal bleeding occurs and not in association with intake of hormone-containing pills. Dysmenorrhea seems to be the most important factor related to negative impact on physical performance by OC users. Although quite many of the athletes already today skip the inactive pills, it could be further recommended to achieve absence of menses and no pain.

The proportion of HC users among those at top-elite level was lower (55%) compared to those at sub-elite level (67%). In this context, the lack of conclusive evidence concerning the impact of HCs on physical and mental performance (Elliott-Sale et al., 2020) might have influenced the willingness of our elite athletes to use HCs. Weight gain is one such potential side effect and 20% of our respondents experienced weight gain, in comparison to approximately 8% reported by Martin et al. (Martin et al., 2018). However, there is no evidence of a causal relationship between combined OCs and weight gain (Gallo et al., 2014).

### 4.1 Limitations and strengths

Like all surveys based on self-reporting, our is subject to recall bias and lack of confirmation of symptoms in relation to the MC or HC use. Although the questionnaire administrated was tested in a pilot study and thereafter modified accordingly, several aspects of its validity and reliability remain to be assessed. In the questionnaire, a picture of the different phases of the MC with an explanatory text was shown to the athletes. However, it is important to be aware of the short time window of the ovulatory phase that may have affected the accuracy of these data.

Since the web-survey was sent out *via* social media and emails the total numbers of receivers is unknown, given the response rate indefinitely. Furthermore, the items concerning refraining from training and/or competition did not specify the duration involved. In addition, our survey did not explore each athlete’s individual history of HC use and possible reason for terminating such usage, which could have provided valuable additional insights. As expected, only a small proportion of the respondents (15%) was classified as top-elite athletes, and this fact might have limited the statistical power of comparison between the different athlete levels. At the same time, our study population was one of the largest analyzed to date (Martin et al., 2018; Findlay et al., 2020; Solli et al., 2020).

## 5 Conclusion

This big dataset shows that menstrual related symptoms are common in female athletes across all athlete levels and often impact their training and performance. However, few athletes schedule their training or competition in relation to these symptoms. Dysmenorrhea and PMS are the most common symptoms and are perceived to negatively relate to different aspects of performance such as aerobic fitness, muscle strength, mental sharpness, balance, and sleep quality. A large proportion of the athletes used HCs, with progestins as the most common method, not only for contraception but also to treat various menstrual related symptoms. Even though side-effects were reported, physical performance such as aerobic fitness, muscle strength, mental sharpness, balance, and sleep quality did not seem to vary during the pill-chart of combined OCs with exception from the time point of inactive pills, which was associated with a negative impact. The results of this survey of 1,086 female athletes indicate that greater focus should be on identifying and effectively treat different MC or HC related symptoms on an individual level.

## Data Availability

The raw data supporting the conclusion of this article will be made available by the authors, without undue reservation.
