# Supplementary material for: Perceived impact of the menstrual cycle and hormonal contraceptives on physical exercise and performance in 1,086 athletes from 57 sports
Source: Front Physiol. 2022 Aug 30;13:954760. doi: 10.3389/fphys.2022.954760 (PMC9468598; doi:10.3389/fphys.2022.954760)
Supplement: Supplementary file 1 [file Table1.docx]

**SUPPLEMENTARY MATERIAL**

**Table 1**. The different brands of combined oral contraceptives (the number of each). The level of ethinyl estradiol and the type and dose of progestin. Estrogen dominant oral contraceptives in the 1st to 3rd row and gestagen dominant oral contraceptives in the 6th to 8th row.

| **Combined OCs (EE* and progestin)** | **Etinyl estradiol**  **(µg/day)** | **Progestin****  **(µg/day)** |
| --- | --- | --- |
| Diane® (7), Zyrona® (2) | 35 | 2000 CPA |
| Yasmin® (11), Rosal® (7), Midiana® (1), Dizmine® (1) | 30 | 3000 DSP |
| Dienorette® (23), Qlaria® (3) | 30 | 2000 DNG |
| Amorest® (11, Zoely® (5), Cilest® (4) | 35 | 250 NGM |
| Daylette®(1), Dizminelle® (1), Estrelen® (14), Estron® (3), Yaz® (1), Yasminelle® (5) | 20 | 3000 DSP |
| Mercilon® (7), Cerazette® (8), Marvelon® (4), Desirett® (4), Gestrina® (2), Desirett® (4) | 20 | 150 DG |
| Trionetta® (1) | 30-40-30 | 50-75-125 LNG |
| Prionelle® (61), Neovletta®/Microgynon® (27), Loette® (16), Leverette® (8), Anastrella® (5), Almina (5), Mirabella® (15), Oralcon® (10), Melleva® (18), Rigevidoncont® (1) | 30 | 150 LNG |
|  |  |  |
|  |  |  |
| **Types of progestin: CPA=cyproteronacetat, DG=desogestrel, DNG=dienogest, DSP=drospirenon, LNG=levonorgestrel, NET=noretisteron, NGM=norgestimat. | |  |
